# LINE-1 hypomethylation is neither present in rectal aberrant crypt foci nor associated with field defect in sporadic colorectal neoplasia

**DOI:** 10.1186/1868-7083-6-24

**Published:** 2014-11-10

**Authors:** Isabel Quintanilla, Maria Lopez-Cerón, Mireya Jimeno, Miriam Cuatrecasas, Jennifer Muñoz, Leticia Moreira, Sabela Carballal, Maria Liz Leoz, Jordi Camps, Antoni Castells, Maria Pellisé, Francesc Balaguer

**Affiliations:** Department of Gastroenterology, Hospital Clínic, Centro de Investigación Biomédica en Red en Enfermedades Hepáticas y Digestivas (CIBEREHD), IDIBAPS, Barcelona, Catalonia, Spain; Pathology Department, Centre de Diagnostic Biomèdic (CDB), Hospital Clínic, University of Barcelona and Banc de Tumors-Biobanc Clinic-IDIBAPS-XBTC, Barcelona, Catalonia, Spain

**Keywords:** Colorectal cancer, Aberrant crypt foci, LINE-1, Methylation, Prevention

## Abstract

**Background:**

Aberrant crypt foci (ACF) are considered the first identifiable preneoplastic lesion in colorectal cancer (CRC), and have been proposed as a potential biomarker for CRC risk. Global DNA hypomethylation is an early event in colorectal carcinogenesis, and long interspersed nuclear element-1 (LINE-1) methylation status is a well-known surrogate marker for genome-wide DNA methylation levels. Despite the gradual increase in DNA hypomethylation in the adenoma–carcinoma sequence, LINE-1 methylation in ACF has never been studied. Moreover, recent studies have reported a field defect for LINE-1 hypomethylation, suggesting that LINE-1 methylation status in normal mucosa could be used to stratify CRC risk and tailor preventive strategies. Thus, we assessed LINE-1 status by pyrosequencing in rectal ACF and paired normal colorectal mucosa from individuals with sporadic colon cancer (CC) (n = 35) or adenoma (n = 42), and from healthy controls (n = 70).

**Findings:**

Compared with normal mucosa, LINE-1 in ACF were hypermethylated across all groups (*P* < 0.0001). Furthermore, LINE-1 methylation status in normal colorectal mucosa was independent of the presence of adenoma or CC (*P* = 0.1072), and did not differ depending on the distance to the adenoma or CC. Interestingly, when we compared the LINE-1 methylation status in normal mucosa from different segments of the colorectum, we found higher hypomethylation in the rectum compared with the descending colon (*P <* 0.0001).

**Conclusions:**

Overall, our results suggest that global hypomethylation is not present in rectal ACF and argues against the existence of LINE-1 methylation field defect in sporadic colon cancer.

**Electronic supplementary material:**

The online version of this article (doi:10.1186/1868-7083-6-24) contains supplementary material, which is available to authorized users.

## Findings

### Background

Aberrant crypt foci (ACF) are considered the first identifiable preneoplastic lesion in CRC [1]. ACF show features that support their potential role as premalignant lesions, such as dysplasia, monoclonality, and mutations, which are present in adenomas and carcinomas but not in normal colon mucosa [2, 3]. High-magnification chromoscopic colonoscopy can detect ACF *in vivo*[4], and several studies using chromoendoscopy have shown a higher rate of ACF occurrence in patients with CRC and adenomas compared with healthy individuals [4]. Accordingly, ACF (especially those located in the rectum) have been proposed as a potential biomarker for CRC risk [5]. Histologically, ACF can be classified as hyperplastic or dysplastic [6]. Data from single-center studies have suggested that dysplastic ACF may correlate with CRC risk, as there is an increased frequency of dysplastic ACF in individuals with a normal colon, patients with adenoma, and patients with CRC [1]. However, other studies have shown conflicting findings regarding the clinical value of dysplastic ACF [7–9], and thus, the role of ACF in colorectal carcinogenesis is still under debate. Although rectal ACF have been suggested as potential biomarkers to identify individuals at high risk for CRC because of their specific histological and molecular features, this is still an area of uncertainty [10].

Methylation of cytosine residues at CpG dinucleotides is a major epigenetic modification strongly associated with transcriptional silencing. Long interspersed element-1 (LINE-1) elements are a type of genomic repetitive elements that in their full-length form encode the retrotransposon machinery [11]. LINE-1 methylation status is a well-known surrogate marker for genome-wide DNA methylation level. In normal cells, LINE-1 is heavily methylated [12], whereas cancer cells show LINE-1 hypomethylation, leading to chromosomal instability [13]. This phenomenon occurs early in the colorectal carcinogenesis [14, 15]. However, despite the gradual increase of hypomethylation in the adenoma–carcinoma sequence [16], LINE-1 methylation in ACF has never been studied. Moreover, recent studies have reported a field defect in LINE-1 hypomethylation [14, 17, 18], suggesting that LINE-1 methylation status in normal mucosa could be used to stratify CRC risk and tailor preventive strategies.

The aims of this study were to analyze the levels of LINE-1 methylation in rectal ACF compared with paired normal colorectal mucosa, and to investigate the putative field defect of LINE-1 hypomethylation by analyzing the normal mucosa of patients from three different CRC risk groups. The analysis was confined to rectal ACF because of their previously reported higher frequency compared with other colonic segments, in addition to their usefulness as CRC biomarkers based on the total number and histological characteristics [14].

## Subjects and methods

### Subjects

**Figure 1:**
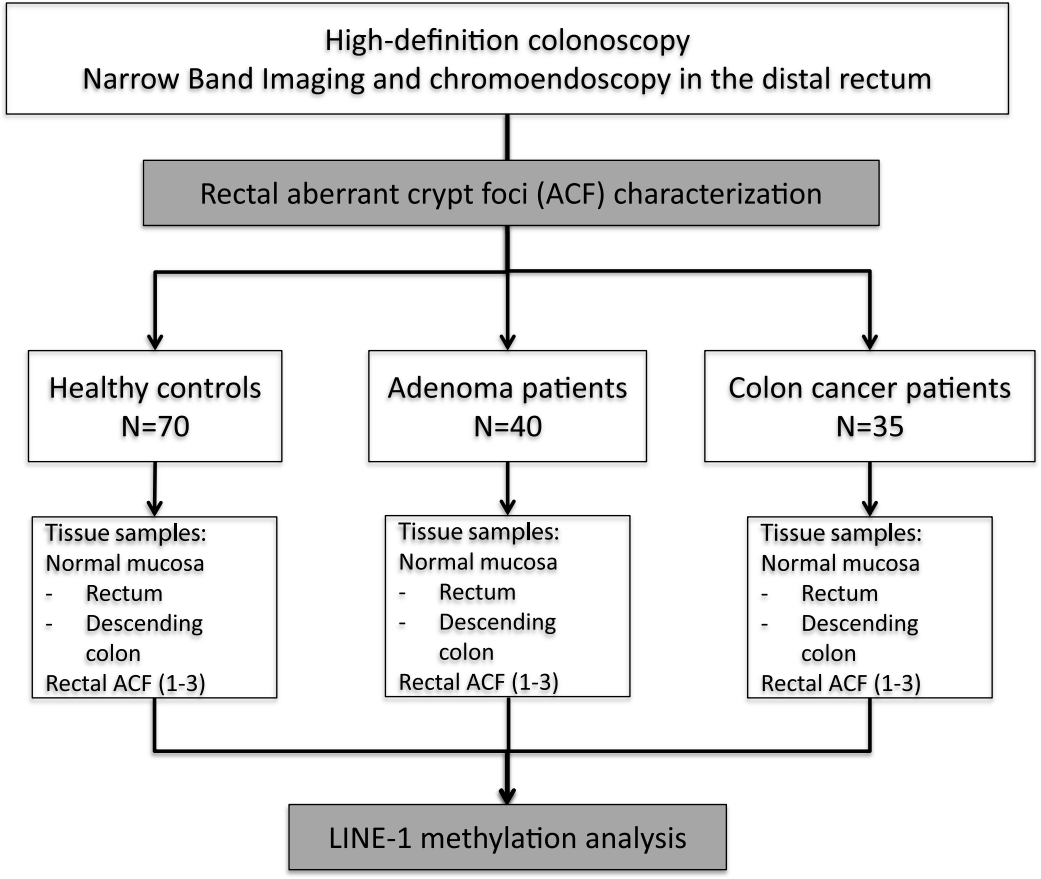
**Study design.** Flow chart summarizing the study design. Long interspersed element-1 (LINE-1) methylation analysis was performed in DNA extracted from aberrant crypt foci (ACF) and normal colorectal mucosa samples from three colon cancer risk groups (individuals with a normal colonoscopy, patients with adenomas, and patients with colon cancer).

A random selection of patients referred to our hospital for a diagnostic colonoscopy between 2008 and 2010 was prospectively included in this study. Colonoscopy was performed with high-definition endoscopes (H180; Olympus, Tokio, Japan). Based on the colonoscopy findings, individuals were classified into healthy control (n = 70), adenoma (n = 42), and colon cancer (CC) (n = 35) groups. The distal rectum (10 cm) was explored with narrow band imaging and then with high-resolution chromoendoscopy using methylene blue 0.5%. For each individual, biopsy samples of normal descending colon and rectal mucosa (with a minimum distance of 10 cm away from any lesion), and up to three ACF in the rectum were used for molecular analyses (Figure 1). Patients with rectal cancer were excluded from the analysis in order to ensure that biopsies were taken at least 10 cm away from the colorectal neoplasia in all patients. Two different endoscopists registered the number and features of ACF, and two expert pathologists characterized all biopsies.

The project was granted approval from institutional review board of the Hospital Clinic of Barcelona, and written informed consent was obtained from all participants.

### DNA extraction

ACF and normal mucosa biopsies were preserved at −80°C optimal cutting compound (OCT) and phosphate-buffered saline (PBS), respectively. DNA was extracted using an All Prep DNA/RNA Mini Kit (catalog number 80204; Qiagen, Hilden, Germany) following the manufacturer’s recommendations. DNA and RNA were quantified using a NanoDrop Spectrophotometer ND-1000 (Thermo Fisher Scientific).

### Bisulfite pyrosequencing and LINE-1 methylation analysis

Bisulfite treatment was performed using the EpiTect Bisulfite kit (catalog number 59104; Qiagen) following manufacturer’s instructions. Methylation analysis of LINE-1 promoter was investigated using pyrosequencing-based methylation analysis [15]. PCR was carried out in a 25 μl PCR mixture containing 12.5 μl of GoTaq Colorless Master Mix (catalog number M7142, Promega, WI, USA) (Reaction Buffer pH 8.5, 400 μM of each dNTP, and 3 mM MgC_l2_), 1 μl of Taq polymerase, 2 μl of forward primer (5′-TTTTGAGTTAGGTGTGGGATATA-3′), 2 μl of reverse biotinylated primer (5′-AAAATCAAAAAATTCCCTTTC-3′), and 100 ng of bisulfite-treated genomic DNA. PCR cycling conditions were 95°C for 15 minutes; 45 cycles of 94°C for 30 seconds, 55°C for 45 seconds, and 72°C for 45 seconds; and finally, 10 minutes at 72°C and 4°C forever. The biotinylated PCR product was purified and made single-stranded to act as a template in a pyrosequencing reaction as recommended by the manufacturer using Pyrosequencing Vacuum Tool (Qiagen, Hilden, Germany). In brief, the PCR product was bound to a Streptavidin Sepharose HP column, and the sepharose beads containing the immobilized PCR product were purified, washed, denatured using 0.2 M NaOH solution, and washed again. Then, 10 μM of pyrosequencing primer (5′AGTTAGGTGTGGGATATAGT-3′) was annealed to the purified single-stranded PCR product, and pyrosequencing was performed using a PSQ 96MA Pyrosequencing System (Qiagen). CpGenome Methylated DNA (catalog number S7821; Millipore, Darmstadt, Germany) was used as a positive control. Methylation level of LINE-1 elements was calculated as the mean percentage of the four CpG sites analyzed, as previously described [19].

### Statistical analysis

Distribution of LINE-1 methylation levels was assessed with the Shapiro–Wilks test, which showed that the data were not normally distributed (*P* = 0.017). Accordingly, the Wilcoxon signed-rank test was used for comparing average methylation levels of paired ACF and normal mucosa. When comparing global methylation between two independent groups and between more than two groups, the Mann–Whitney *U*-test and Kruskal–Wallis test, respectively, were used. LINE-1 methylation levels are expressed as median and interquartile range (IQR). All statistical analyses were performed using SPSS software (v20; IBM Inc., Armonk, NY, USA) and results were considered significant at *P <*0.05.

## Results

Baseline characteristics of patients and rectal ACF related to CRC risk group are detailed in Table 1. As shown, the adenoma group mainly comprised patients with advanced adenomas (mean size 12.3 ± 9.8 mm) located throughout the colorectum. Although 16 patients (40%) with adenoma had synchronous serrated polyps, the vast majority of them were small hyperplastic polyps located in the rectosigmoid colon, which are known to lack clinical significance.

**Table 1:** **Baseline characteristics of individuals and aberrant crypt foci in each colon cancer risk group**

	Control ( n = 70)	Adenoma ( n = 42)	Colon cancer ( n = 35)
Sex, n (%)			
– Female	48 (68.6%)	17 (40.5%)	13 (37.1%)
– Male	22 (31.4%)	25 (59.5%)	22 (62.9%)
Age, years, mean ± SD	58.1 ± 15.3	68.6 ± 9.9	66.8 ± 8.6
Colon cancer location, n (%)			
– Sigmoid	–	–	21 (60%)
– Descending	–	–	2 (5.7%)
– Transverse	–	–	1 (2.9%)
– Ascending	–	–	9 (25.7%)
– Cecum	–	–	2 (5.7%)
Colon cancer TNM stage, n (%)			
– I	–	–	10 (28.6%)
– II	–	–	11 (31.4%)
– III	–	–	13 (37.1%)
– IV	–	–	1 (2.9%)
Adenoma features (per patient)^a^			
– Tubular, n (%)	–	28 (70%)	11 (31.4%)
– Tubulovillous, n (%)	–	7 (17.5%)	1 (2.9%)
– Villous, n (%)	–	5 (12.5%)	0 (0%)
– High-grade dysplasia, n (%)	–	11 (27.5%)	5 (14.3%)
– Adenoma size, mean (mm) ± SD	–	12.3 ± 9.8	8.4 ± 5
– Number of adenomas per patient, mean ± SD (range)	–	2.4 ± 2 (1 to 9)	1.2 ± 1.2 (1 to 4)
Advanced adenoma^b^, n (%)		22 (55%)	7 (20%)
Adenoma location (per patient)^c^, n (%)			
– Rectum	–	12 (30%)	2 (5.7%)
– Sigmoid	–	10 (25%)	3 (8.6%)
– Descending	–	5 (12.5%)	3 (8.6%)
– Transverse	–	5 (12.5%)	2 (5.7%)
– Ascending	–	5 (12.5%)	2 (5.7%)
– Cecum	–	3 (7.5%)	0 (0%)
Serrated polyps features (per patient)^d^			
– Recto-sigmoid hyperplastic polyps, n (%)	–	12 (30%)	3 (8.6%)
– Proximal serrated polyps, n (%)^e^	–	4 (10%)	5 (14.3%)
– Serrated polyps ≥10 mm, n (%)	–	2 (5%)	2 (5.7%)
– Serrated polyps per patient, n, mean ± SD (range)	–	1.6 ± 0.8 (1 to 3)	3.1 ± 3.4 (1 to 5)
– Hyperplastic polyp, n (%)	–	15 (37.5%)	8 (22.9%)
– Sessile serrated adenoma, n (%)	–	1 (%)	0 (0%)
–Traditional serrated adenoma, n (%)	–	0 (%)	0 (0%)
ACF for molecular study, n	117	76	77
Dysplastic ACF, n (%)	24 (17.4%)	23 (24%)	17 (25%)
Hyperplastic ACF, n (%)	114 (82.6%)	73 (76%)	51 (75%)

ACF, aberrant crypt foci; SD, standard deviation.

^a^This referred to 40 individuals in the adenoma group for whom full pathological information about the adenomas was available.

^b^Advanced adenoma: ≥10 mm in size, or presence of high-grade dysplasia or villous features.

^c^In cases with >1 adenoma, this referred to the location of the most advanced adenoma.

^d^Included hyperplastic polyps, sessile serrated adenomas, and traditional serrated adenomas.

^e^Proximal to the sigmoid colon.

### Rectal ACF do not display LINE-1 hypomethylation

**Figure 2:**
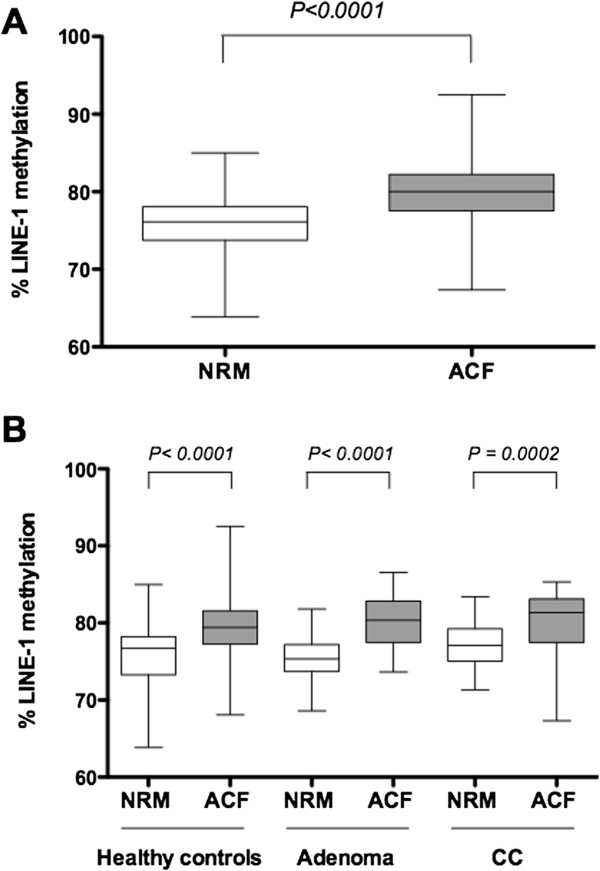
**Long interspersed element-1 (LINE-1) methylation status in aberrant crypt foci (ACF) and normal rectal mucosa. (A)** LINE-1 methylation levels in ACF compared with normal rectal mucosa (NRM) samples. **(B)** ACF LINE-1 methylation levels compared with NRM samples stratified by risk group. Box-and-whisker plot indicating the median methylation level expressed as a percentage (horizontal line), 25th and 75th percentiles (box), and maximum and minimum levels (whiskers).

To test the role of rectal ACF as a potential precursor lesion, we analyzed the LINE-1 methylation levels in rectal ACF and paired normal rectal and descending colon mucosa from all patients. The median LINE-1 methylation level of up to three ACF for each patient was considered for statistical purposes using the Wilcoxon signed-rank test. As shown in Figure 2A, our data indicated that overall, levels of LINE-1 methylation were higher in ACF than in normal rectal mucosa (80.02% (77.54 to 82.22) versus 76.13% (73.74 to 78.06); *P* < 0.0001). In fact, only 20 subjects exhibited lower LINE-1 methylation in their rectal ACF compared with their normal mucosa, and LINE-1 in rectal ACF was not hypomethylated in any of the three risk groups (Figure 2B). Likewise, similar results were observed for LINE-1 methylation levels when comparing rectal ACF and normal mucosa from descending colon [80.02% (77.54 to 82.22) versus 78.42% (76.51 to 80.33]; *P* = 0.0002) (see Additional file 1: Figure S1). We also performed these analyses stratifying by sex, with similar results for both males and females (see Additional file 2: Table S1). Interestingly, there were significant differences in LINE-1 methylation between dysplastic ACF and hyperplastic ACF [81.46% (78.75 to 82.97) versus 79.54% (76.88 to 81.79); *P =* 0.031].

### LINE-1 methylation levels in normal colorectal mucosa are similar in all CC risk groups

**Figure 3:**
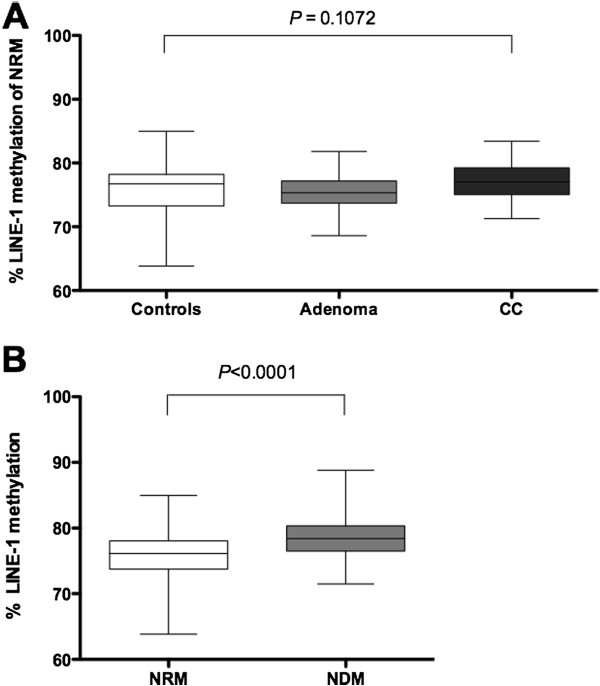
**Evaluation of long interspersed element-1 (LINE-1) methylation in normal colorectal mucosa as a potential field defect. (A)** LINE-1 methylation levels in normal rectal mucosa (NRM) stratified by colon cancer (CC) risk group. **(B)** LINE-1 methylation levels in NRM compared with normal mucosa from the descending colon (NDM). Box-and-whisker plot indicating the median methylation level expressed as a percentage (horizontal line), 25th and 75th percentile (box), and maximum and minimum levels (whiskers).

To assess the LINE-1 field defect, we compared the methylation status of LINE-1 in the normal rectal and descending colon mucosa of individuals from the three CC risk groups (CC, adenoma and healthy controls). Because the healthy control group was significantly younger than the adenoma and CC groups (Table 1), we first excluded an age effect in the level of LINE-1 methylation in normal mucosa (see Additional file 3: Figure S2). As shown in Figure 3A, methylation of LINE-1 in normal rectal mucosa was independent of the patient group [healthy controls: 76.73% (73.23 to 78.22); adenoma: 75.34% (73.7 to 77.2); CC: 77.06% (75.04 to 79.23); *P =* 0.107] (Table 2). Comparable results were obtained when normal mucosa from the descending colon was considered [healthy controls: 77.67% (75.84 to 80.18); adenoma: 78.45% (77.37 to 79.92); CC: 80.30% (77.19 to 81.39); *P =* 0.147] (Table 2). These analyses were also performed with stratification by sex, with similar results (see Additional file 2: Table S1).

**Table 2:** **LINE-1 methylation levels in normal mucosa and aberrant crypt foci for each of the three groups of subjects**

	Healthy controls (n = 70)	Adenoma (n = 42)	Colon cancer (n = 35)	***P*** value ^a^
Normal rectal mucosa, n/N	62/70	31/42	24/35	0.107
Median (IQR)	76.73% (73.23 to 78.22)	75.34% (73.7 to 77.2)	77.06% (75.04 to 79.23)	
Normal descending colon mucosa, n/N	42/70	38/42	21/35	0.147
Median (IQR)	77.67% (75.84 to 80.18)	78.45% (77.37 to 79.92)	80.30% (77.19 to 81.39)	
Aberrant crypt foci, n/N	68/70	38/42	24/35	0.409
Median (IQR)	79.41% (77.24 to 81.58)	80.32% (77.45 to 82.82)	81.37% (77.48 to 83.14)	
Dysplastic aberrant crypt foci, n/N	15	11	9	0.32
Median (IQR)	80.58% (78.54 to 82.37)	80.99% (79.15 to 83.07)	82.74% (79.83 to 83.43)	
Hyperplastic aberrant crypt foci, n/N	53	27	24	0.812
Median (IQR)	79.32% (76.94 to 81.54)	79.59% (76.80 to 82.22)	80.02% (76.50 to 82.55)	

IQR, interquartile range; n, number of samples analyzed in each group; N, total number of samples included in each group.

^a^Kruskal–Wallis test was used to assess the level of LINE-1 methylation between the three risk groups.

We also analyzed the potential field defect for LINE-1 methylation by stratifying the results by the distance of the lesion to the normal mucosa biopsies. As shown in Table 3, LINE-1 methylation levels in normal mucosa did not differ depending on the distance to the adenoma or CC.

**Table 3:** **LINE-1 methylation levels in normal mucosa stratifying by location of the neoplastic lesion**

LINE-1 methylation	Patients with proximal adenoma (n = 10) ^a^	Patients with distal adenoma (n = 30) ^a^	***P*** value	Patients with proximal ^a^ colon cancer (n = 11)	Patients with distal colon cancer (n = 24) ^a^	***P*** value
Normal rectal mucosa	73.87% (72.47 to 75.90)	76.06% (74.78 to 77.33)	0.087	77.59% (72.78 to 80.12)	77.40% (75.70 to 79.79)	0.794
Normal descending mucosa	80.72% (76.62 to 82.68)	78.38% (76.55 to 80.01)	0.176	77.14% (75.59 to 80.06)	78.59% (76.90 to 79.41)	0.458

LINE-1 methylation levels are expressed as median (interquartile range, IQR).

^**a**^Referred to the splenic flexure.

### LINE-1 methylation levels in normal mucosa differ between colonic segments

Interestingly, when we compared the LINE-1 methylation status in normal mucosa from different segments of the colorectum, we found a higher degree of hypomethylation in the rectum compared with the descending colon [76.13% (73.75 to 78.06) versus 78.42% (76.51 to 80.33); *P <* 0.0001], suggesting different genome-wide methylation profiles between normal colon and rectal mucosa (Figure 3B). Stratification by sex did not vary the results (see Additional file 2: Table S1).

## Discussion

Overall, our results argue against the hypothesis that global hypomethylation occurs at the earliest stages of colorectal carcinogenesis. Overexpression of β-catenin and *KRAS* mutations may indicate that ACF constitute a premalignant stage [16]. Moreover, LINE-1 hypomethylation is already present at the adenoma stage [15]. Our data suggest, for the first time, to our knowledge, that the formation of ACF is not triggered by hypomethylation in LINE-1 elements, and that genome-wide hypomethylation occurs after ACF formation, most likely during the transition from non-adenomatous to adenomatous epithelia. In fact, hypermethylation of LINE-1 elements in ACF suggests that reversal epigenetic changes occur in these lesions regardless of their pathogenic progression.

Despite the fact that two previous reports have suggested the existence of LINE-1 hypomethylation field defects in CRC, our results indicate the absence of this phenomenon in our sample set. Kamiyama and collaborators recently showed that normal colonic mucosa from patients with synchronous CRC displayed higher hypomethylation of LINE-1 compared with patients with solitary CRC and controls [17]. However, and in line with our results, there were no differences between the two latter groups. Another study has shown that normal colorectal mucosa samples from different risk groups displayed differences in LINE-1 methylation that mirrored differences between their respective tumor specimens [17]. A more recent study has shown that adjacent mucosa from individuals with multiple/large serrated polyps display significantly lower LINE-1 methylation levels compared with individuals without such polyps [18]. We tested the hypothesis that LINE-1 methylation in normal mucosa could be used as a biomarker for risk of sporadic CRC, focusing on patients with conventional adenomas. By analyzing a prospective cohort of patients with CC and controls, we found that our data suggest that methylation status of LINE-1 in normal mucosa is not a suitable biomarker to predict CC risk in patients with sporadic cancer. However, the potential field defect for LINE-1 methylation in patients with serrated polyps needs further assessment. In addition, our data show different methylation levels in normal mucosa between the descending colon and the rectum, suggesting different methylation profiling depending on the colonic segment.

## Conclusion

Our results shed some light on the role of genome-wide methylation in rectal ACF and argue against its utility as a biomarkers for assessing CC risk.

## Authors’ information

MP and FB share senior authorship.

## Electronic supplementary material

Additional file 1: Figure S1: LINE-1 methylation status in aberrant crypt foci and normal mucosa from the descending colon. (A) LINE-1 methylation levels in aberrant crypt foci (ACF) compared to normal mucosa from the descending colon (NDM). (B) ACF LINE-1 methylation levels compared to descending mucosa samples according to risk group. Box-and-whisker plot indicating the median methylation level expressed as a percentage (horizontal line), 25^th^ and 75^th^ percentile (box), and maximum and minimum levels (whiskers). (TIFF 3 MB)

Additional file 2: Table S1: LINE-1 methylation analysis stratified by sex. (DOCX 46 KB)

Additional file 3: Figure S2: Evaluation of the age-effect for LINE-1 methylation data. Representation of the correlation between LINE-1 methylation levels in normal rectal mucosa and the age of patients. (TIFF 4 MB)

## Abbreviations

ACF: Aberrant crypt foci
CC: Colon cancer
CRC: Colorectal cancer
LINE-1: Long interspersed element-1
NDM: Normal descending mucosa
NRM: Normal rectal mucosa

## References

[1] Takayama T, Katsuki S, Takahashi Y, Ohi M, Nojiri S, Sakamaki S, Kato J, Kogawa K, Miyake H, Niitsu Y (1998). Aberrant crypt foci of the colon as precursors of adenoma and cancer. N Engl J Med.

[2] McLellan EA, Medline A, Bird RP (1991). Dose response and proliferative characteristics of aberrant crypt foci: putative preneoplastic lesions in rat colon. Carcinogenesis.

[3] Bird RP, Good CK (2000). The significance of aberrant crypt foci in understanding the pathogenesis of colon cancer. Toxicol Lett.

[4] Yokota T, Sugano K, Kondo H, Saito D, Sugihara K, Fukayama N, Ohkura H, Ochiai A, Yoshida S (1997). Detection of aberrant crypt foci by magnifying colonoscopy. Gastrointest Endosc.

[5] Gupta S, Ashfaq R, Kapur P, Afonso BB, Nguyen TP, Ansari F, Boland CR, Goel A, Rockey DC (2011). Microsatellite instability among individuals of Hispanic origin with colorectal cancer. Cancer.

[6] Carr NJ SL, Neiderau C (2000). Aberrrant crypt foci. Pathology and genetics: Tumors of the digestive system. WHO Classification of Tumours Series.

[7] Adler DG, Gostout CJ, Sorbi D, Burgart LJ, Wang L, Harmsen WS (2002). Endoscopic identification and quantification of aberrant crypt foci in the human colon. Gastrointest Endosc.

[8] Pretlow TP, Pretlow TG (2005). Mutant KRAS in aberrant crypt foci (ACF): initiation of colorectal cancer?. Biochim Biophys Acta.

[9] Suehiro Y, Hinoda Y (2008). Genetic and epigenetic changes in aberrant crypt foci and serrated polyps. Cancer Sci.

[10] Mutch MG, Schoen RE, Fleshman JW, Rall CJ, Dry S, Seligson D, Charabaty A, Chia D, Umar A, Viner J, Hawk E, Pinsky PF (2009). A multicenter study of prevalence and risk factors for aberrant crypt foci. Clin Gastroenterol Hepatol.

[11] Speek M (2001). Antisense promoter of human L1 retrotransposon drives transcription of adjacent cellular genes. Mol Cell Biol.

[12] Kazazian HH (2004). Mobile elements: drivers of genome evolution. Science.

[13] Howard G, Eiges R, Gaudet F, Jaenisch R, Eden A (2008). Activation and transposition of endogenous retroviral elements in hypomethylation induced tumors in mice. Oncogene.

[14] Pavicic W, Joensuu EI, Nieminen T, Peltomaki P (2012). LINE-1 hypomethylation in familial and sporadic cancer. J Mol Med (Berl).

[15] Sunami E, de Maat M, Vu A, Turner RR, Hoon DS (2011). LINE-1 hypomethylation during primary colon cancer progression. PLoS One.

[16] Ibrahim AE, Arends MJ, Silva AL, Wyllie AH, Greger L, Ito Y, Vowler SL, Huang TH, Tavare S, Murrell A, Brenton JD (2011). Sequential DNA methylation changes are associated with DNMT3B overexpression in colorectal neoplastic progression. Gut.

[17] Kamiyama H, Suzuki K, Maeda T, Koizumi K, Miyaki Y, Okada S, Kawamura YJ, Samuelsson JK, Alonso S, Konishi F, Perucho M (2012). DNA demethylation in normal colon tissue predicts predisposition to multiple cancers. Oncogene.

[18] Yamada A, Minamiguchi S, Sakai Y, Horimatsu T, Muto M, Chiba T, Boland CR, Goel A (2014). Colorectal advanced neoplasms occur through dual carcinogenesis pathways in individuals with coexisting serrated polyps. PLoS One.

[19] Antelo M, Balaguer F, Shia J, Shen Y, Hur K, Moreira L, Cuatrecasas M, Bujanda L, Giraldez MD, Takahashi M, Cabanne A, Barugel ME, Arnold M, Roca EL, Andreu M, Castellvi-Bel S, Llor X, Jover R, Castells A, Boland CR, Goel A (2012). A high degree of LINE-1 hypomethylation is a unique feature of early-onset colorectal cancer. PLoS One.

