# Supplementary figures and images for: LINE-1 hypomethylation is neither present in rectal aberrant crypt foci nor associated with field defect in sporadic colorectal neoplasia
Source: Clin Epigenetics. 2014 Nov 10;6(1):24. doi: 10.1186/1868-7083-6-24 (PMC4391726; doi:10.1186/1868-7083-6-24)

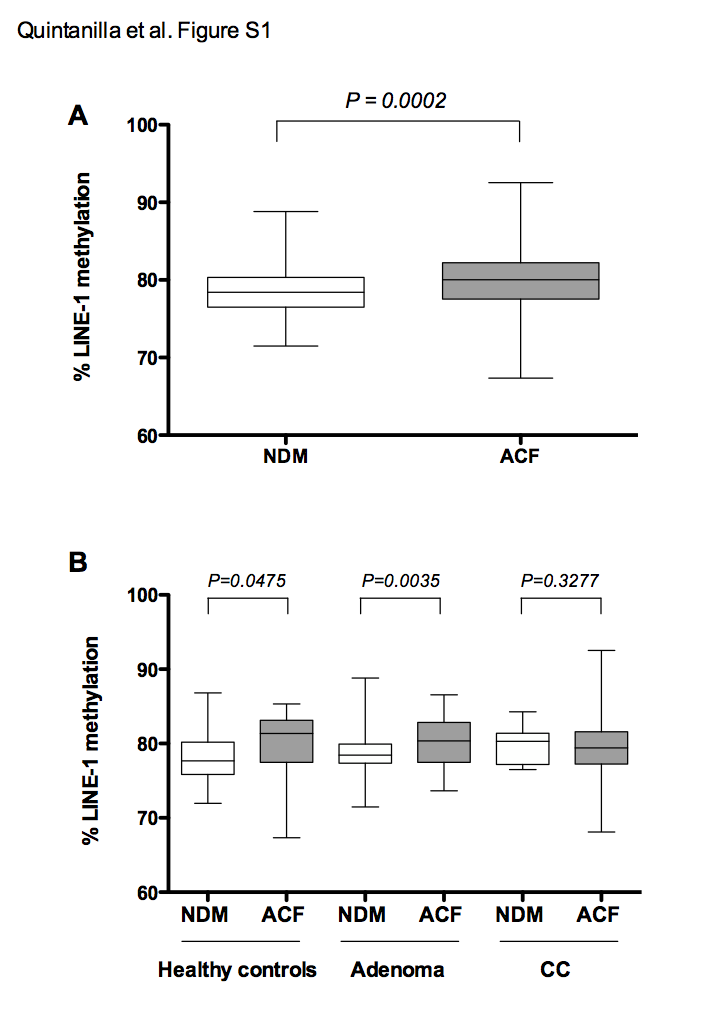

Supplement: Supplementary file 1 — Additional file 1: Figure S1: LINE-1 methylation status in aberrant crypt foci and normal mucosa from the descending colon. (A) LINE-1 methylation levels in aberrant crypt foci (ACF) compared to normal mucosa from the descending colon (NDM). (B) ACF LINE-1 methylation levels compared to descending mucosa samples according to risk group. Box-and-whisker plot indicating the median methylation level expressed as a percentage (horizontal line), 25th and 75th percentile (box), and maximum and minimum levels (whiskers). (TIFF 3 MB) [file 13148_2014_95_MOESM1_ESM.tiff]

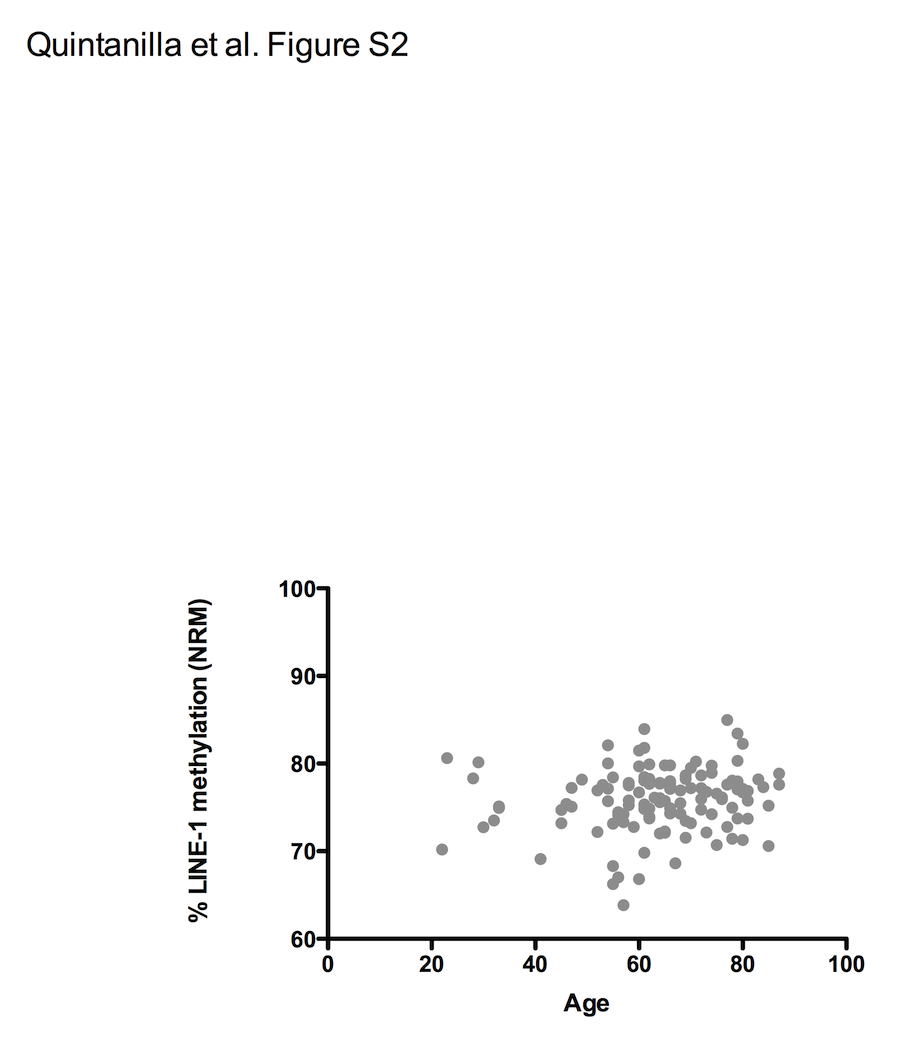

Supplement: Supplementary file 3 — Additional file 3: Figure S2: Evaluation of the age-effect for LINE-1 methylation data. Representation of the correlation between LINE-1 methylation levels in normal rectal mucosa and the age of patients. (TIFF 4 MB) [file 13148_2014_95_MOESM3_ESM.tiff]
