# Supplementary material for: LINE-1 hypomethylation is neither present in rectal aberrant crypt foci nor associated with field defect in sporadic colorectal neoplasia
Source: Clin Epigenetics. 2014 Nov 10;6(1):24. doi: 10.1186/1868-7083-6-24 (PMC4391726; doi:10.1186/1868-7083-6-24)
Supplement: Supplementary file 2 — Additional file 2: Table S1: LINE-1 methylation analysis stratified by sex. (DOCX 46 KB) [file 13148_2014_95_MOESM2_ESM.docx]

**Additional file 2: Table S1. LINE-1 methylation analysis stratified by gender**

|  | **Gender** | **N** | **Median (IQR)** | **p value** |
| --- | --- | --- | --- | --- |
| **LINE-1 methylation in normal rectal mucosa versus ACF** |  |  |  |  |
| - Overall | Male | 53 vs 62 | 76.76 (74.25-78.55) vs 80.32 (77.51-82.39) | p < 0.0001 |
|  | Female | 64 vs72 | 75.69 (73.33-77.95) vs 79.72 (77.08-82.00) | p < 0.0001 |
| - Healthy controls | Male | 19 vs 22 | 76.95 (73.50-80.02) vs 80.19 (77.00-81.55) | p = 0.0207 |
|  | Female | 43 vs 46 | 76.11 (72.74-78.00) vs 79.33 (77.59-81.78) | p < 0.0001 |
| - Adenoma | Male | 18 vs 22 | 74.87 (73.10-77.21) vs 79.93 (77.37-82.36) | p = 0.0005 |
|  | Female | 13 vs 16 | 74.40 (73.72-77.41) vs 80.79 (77.32-83.91) | p = 0.0105 |
| - Colon cancer | Male | 16 vs 18 | 77.60 (76.02-79.80) vs 81.73 (77.51-84.51) | p = 0.0026 |
|  | Female | 8 vs 10 | 75.63 (73.27-79.91) vs 80.07 (74.33-82.34) | p = 0.1563 |
| **LINE-1 methlation in normal descending mucosa versus ACF** |  |  |  |  |
| - Overall | Male | 49 vs 62 | 78.44 (76.42-80.66) vs 80.32 (77.51-82.39) | p = 0.1031 |
|  | Female | 54 vs 72 | 78.07 (76.34-80.28) vs 79.72 (77.08-82.01) | p = 0.0320 |
| - Healthy controls | Male | 22 vs 11 | 78.90 (77.13-81.23) vs 80.19 (77.00-81.55) | p = 0.3203 |
|  | Female | 31 vs 46 | 77.64 (75.33-79.66) vs 79.33 (77.59-81.78) | p = 0.0155 |
| - Adenoma | Male | 23 vs 22 | 78.44 (76.52-80.72) vs 79.93 (77.37-82.36) | p = 0.6143 |
|  | Female | 14 vs 16 | 79.13 (77.49-80.69) vs 80.79 (77.32-83.91) | p = 0.4543 |
| - Colon cancer | Male | 15 vs18 | 78.40 (75.87-79.41) vs 81.73 (77.51-84.51) | p = 0.0923 |
|  | Female | 8 vs 10 | 78.19 (76.72-80.15) vs 80.07 (74.33-82.34) | p = 0.8438 |
| **LINE-1 methylation according to ACF histology** |  |  |  |  |
| - Hyperplastic ACF vs dysplastic ACF | Male | 47 vs 15 | 79.66 (77.06-82.01) vs 81.54 (79.15-83.07) | p = 0.0936 |
|  | Female | 54 vs 18 | 79.33 (76.30-81.60) vs 81.02 (78.70-82.52) | p = 0.1453 |
| **LINE-1 methylation in normal rectal mucosa** |  |  |  |  |
| - Healthy controls vs Adenoma vs CC | Male | 19 vs 18 vs 16 | 76.95 (73.50-80.02) vs 74.87 (73.10-77.20) vs 77.60 (76.02-79.80) | p = 0.0510 |
|  | Female | 43 vs 13 vs 8 | 76.11 (72.74-78.00) vs 75.40 (73.72-77.41) vs 75.63 (73.27-79.91) | p = 0.8400 |
| **LINE-1 methylation in normal mucosa from descending colon** |  |  |  |  |
| - Healthy controls vs Adenoma vs CC | Male | 11 vs 23 vs 15 | 78.90 (77.13-81.23) vs 78.44 (76.52-80.72) vs 78.40 (75.87-79.41) | p = 0.6920 |
|  | Female | 31 vs 15 vs 8 | 77.64 (75.33-79.66) vs 79.13 (77.49-80.69) vs 78.19 (76.72-80.15) | p = 0.2010 |
| **LINE-1 methylation in normal mucosa from different colonic segments** |  |  |  |  |
| - Normal rectal mucosa vs normal mucosa from descending colon | Male | 53 vs 49 | 76.76 (74.25-78.55) vs 78.44 (76.42-80.66) | p = 0.0037 |
|  | Female | 64 vs 54 | 75.69 (73.33-77.95) vs 78.07 (76.34-80.28) | p = 0.0002 |
| **LINE-1 methylation in ACF among CC risk groups** |  |  |  |  |
| - ACF Healthy controls vs adenoma vs CC | Male | 22 vs 22 vs 18 | 80.19 (77.00-81.55) vs 79.93 (77.37-82.36) vs 81.73 (77.51-84.51) | p = 0.3624 |
|  | Female | 46 vs 46 vs 10 | 79.33 (77.59-81.78) vs 80.79 (77.32-83.91) vs 80.07 (74.33-82.34) | p = 0.6666 |
| **LINE-1 methylation in ACF stratified by gender** |  |  |  |  |
| - Healthy controls (Male vs Female) |  | 22 vs 46 | 80.19 (77.00-81.55) vs 79.33 (77.59-81.78) | p = 0.9686 |
| - Adenoma (Male vs Female) |  | 22 vs 16 | 79.93 (77.37-82.36) vs 80.79 (77.32-83.01) | p = 0.7562 |
| - Colon cancer (Male vs Female) |  | 18 vs 10 | 81.73 (77.51-84.51) vs 80.07 (74.33-82.34) | p = 0.2401 |

IQR: interquartile range; ACF: aberrant crypt foci; CC: colon cancer
